# A Reliable Data Delivery Mechanism for Grid Power Quality Using Neural Networks in Wireless Sensor Networks

**DOI:** 10.3390/s101009349

**Published:** 2010-10-18

**Authors:** Yujin Lim, Hak-Man Kim, Sanggil Kang

**Affiliations:** 1 Department of Information Media, University of Suwon/San 2-2, Wau-ri, Bongdam-eup, Hwaseong-si, Gyeonggi-do, 445-743, Korea; E-Mail: yujin@suwon.ac.kr; 2 Department of Electrical Engineering, University of Incheon/12-1, Sondo-dong, Yeonsu-gu, Incheon, 406-840, Korea; 3 Computer Science and Information Engineering, Inha University/253, Yonghyun-dong, Nam-gu, Incheon, 402-751, Korea; E-Mail: sgkang@inha.ac.kr

**Keywords:** neural network, sensor network, cost function, data delivery mechanism, power quality

## Abstract

Power grids deal with the business of generation, transmission, and distribution of electric power. Current systems monitor basic electrical quantities such as voltage and current from major pole transformers using their temperature. We improve the current systems in order to gather and deliver the information of power qualities such as harmonics, voltage sags, and voltage swells. In the system, data delivery is not guaranteed for the case that a node is lost or the network is congested, because the system has in-line and multi-hop architecture. In this paper, we propose a reliable data delivery mechanism by modeling an optimal data delivery function by employing the neural network concept.

## 1. Introduction

Power grids involve generation, transmission and distribution of electric power. The electrical distribution system delivers electric power through feeders and pole transformers from distribution substations to end users such as houses, office buildings, and factories. Power quality is any power problem manifested as voltage, current, or frequency deviations, that results in failure or malfunctioning of the customer’s equipment [1].

In general, current systems monitor basic electrical quantities such as voltage and current from major pole transformers using their temperature. For evaluating the current status of power quality, finding places where power quality problems are occurring, and planning measures, we need additional information about power quality. We improve the current systems in order to gather and deliver power quality information parameters such as harmonics, voltage sags, and voltage swells.

To expand the power quality monitoring system, there are various issues such as measurements, controls, databases, and communications. In order to design the communication network, wireless multi-hop communication paradigm is often employed to construct an electrical distribution system (EDS) to reduce the deployment and management costs. Many studies have paid attention to building EDS using wireless sensor networks (WSNs) [2,3]. The reason for using a WSN [4–8] is its efficiency in monitoring numerous computing and sensing devices distributed within a large-scale environment.

A WSN for a power quality monitoring system delivers power quality information generated by pole transformers to a remote monitoring center in the residential division. Usually, the power quality information is periodically measured, gathered, and transmitted to the monitoring center. Once power quality measured at a pole transformer is out of a normal range, an alarm message with detailed contents is promptly sent in the event based manner. In the system, data delivery is not guaranteed in the case that a node is lost or the network is congested, because the system has in-line and multi-hop architecture.

To solve the problem, we propose a reliable data delivery mechanism by modeling an optimal data delivery function. The performance of the function lies in determining the optimal coefficients in the function considering the wireless propagation environment or the topological environment around the node. To do that, we employ the neural network (NN) concept [9].

The remainder of this paper is structured as follows. Section 2 describes our system architecture. Section 3 explains our data delivery mechanism. Following this, we verify the designed system by NS-2 simulations in Section 4. Finally, Section 5 summarizes our results, discusses our future plans, and offers conclusions.

## 2. System Architecture

**Figure 1.**
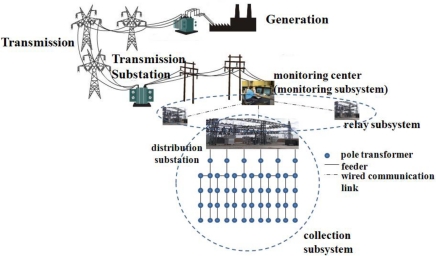
Network infrastructure for EV charging.

An EDS can have tens of thousands of pole transformers ranging widely over hundreds of square kilometers. A monitoring center in a residential division of a city is a data collecting point which gathers the power quality information from scattered pole transformers deployed over the city. The distribution network for an EDS consists of three subsystems, as shown in Figure 1; a collection subsystem, a relay subsystem, and a monitoring subsystem. The collection subsystem is composed of several distribution substations (hereafter, the term ‘substation’ is exchangeable with ‘distribution substation’). Each substation is connected to several feeders. Each feeder collects the power quality data from hundreds of pole transformers and delivers them to the substation. Since pole transformers have been deployed sparsely at distances of hundred meters, a WSN using the IEEE 802.11b standard [10] is employed to construct the collection subsystem in order to reduce the deployment and management costs. The relay subsystem is responsible for delivering the data gathered by the substations to the monitoring subsystem via wired infrastructure due to the long distance between the relay subsystem and the monitoring subsystem. The monitoring center in the monitoring subsystem processes the power quality data to recognize the current status of situations and takes appropriate actions based on the assessed situation [11]. Since substations in the relay subsystem are connected to the monitoring center through a high-speed wired network, the communication between them is highly reliable. Thus the problem of data delivery in EDS is the same as the data delivery problem at the collection subsystem.

## 3. Data Delivery Mechanism

### 3.1. Path Construction and Data Forwarding Mechanism

In EDS, all pole transformers (hereafter, we will use the term ‘node’) can be data sources, while the monitoring center alone is a data sink. In addition, the network topology in EDS is stationary. We design a reliable data forwarding protocol for the collection subsystem.

Since the packet loss probability in wireless multi-hop communication environment increases with the number of hops [12], we choose the Hop Distance (HD) from the node to the substation as one of the metrics for path management. Besides, it is well known that packet loss is due to either collisions or weak signals [13]. By exchanging HELLO messages among nodes, each node measures Received Signal Strength (RSS) and HELLO Message Reception Ratio (HMRR) of its neighbor nodes. HMRR represents the ratio of the number of HELLO message received from a neighbor node to the number of the Hello message sent by the node. We assume that wireless channel is symmetric and HMRR reflects the impact of channel contention from neighbor nodes. Finally, in order to reflect the degree of congestion of a node, Queue Length (QL) of each node is also employed as one of cost factors and QL is included and delivered in HELLO message.

At the network initialization stage, the substation floods a PROBE message over the entire network so that each node in the network can infer the minimum number of hops from the substation to itself. Thereafter, the substation floods a PROBE message periodically so that nodes can update their hop distance from the substation. The path cost is used in constructing the path between the substation and one node. For example, when node *A* receives a PROBE message, it increases the path cost in the message by 1 and compares the increased cost with its path cost. If its path cost is larger than the increased path cost, it updates its path cost with the increased cost. And it configures the PROBE message sending node as its parent node and forwards the PROBE message with its path cost. Otherwise, node *A* configures the PROBE message sending node as its child node and drops the message.

A node periodically sends a HELLO message including its QL to its neighboring node as its heartbeat. When a node receives a HELLO message, the node updates the soft state on the node having sent. If a node or the wireless link to the node fails, any HELLO messages from the node are not arrived for a given amount of time. Thus, the soft state on the node is released. The node detecting the node failure tries to repair the broken path by sending a REPAIR message to its neighboring nodes via the one-hop flooding. Once a neighboring node receives the REPAIR message, it responds with a REPAIR_ACK message having its path cost. Then, the node having sent the REPAIR message receives the REPAIR_ACK message(s) and it selects the node having the least path cost as its next node towards the monitoring center. To select the next-hop node, it is important to determine an optimal link cost function.

Once the data forwarding path is constructed, the power quality data is delivered to the monitoring center through the path. Whenever a node has data to send, periodically or in the event-based manner, the node transmits the data to its next node. This forwarding process continues until the monitoring center receives the power quality information.

### 3.2. Modeling Cost Function by Employing NN Concept

The link cost function depends on the input features based on the characteristics of wireless propagation, channel contention, and topological environment surrounding a node such as HMRR denoted as *x*_1_, QL denoted as *x*_2_, RSS denoted as *x*_3_, and HD denoted as *x*_4_. We also generalize the number of inputs during derivation of the cost function because the number of inputs is varied according to applications.

**Figure 2.**
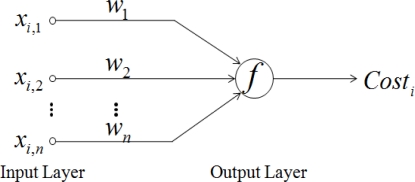
Link cost function represented in a two-layered neural network.

The link cost function can be characterized as a nonlinear function of a weighted sum of the inputs as seen in Equation (1). In general, each weight value is determined by the importance of the corresponding input:

(1) $${\mathrm{Cost}}_{i}=f(X_{i}\cdot W^{T})$$

where *Cost_i_* is the link cost of the *i*th neighbor node out of *N* neighbor nodes and *f* is a nonlinear function. *X_i_* is the input vector collected from the *i*th neighbor node, composed of [*x_i_*_,1_, *x_i,_*_2_,…, *x_i,n_*] (*n* is the number of inputs) and *W* is the corresponding weight vector composed of [*w*_1_,*w*_2_,…,*w_n_*]. Also, *T* is the notation of vector transpose. For fair comparison in the function, we normalize each input into the range in [0, 1] using min. and max. value of each input samples. The equation can be represented in a two layered NN in which the input layer consisted of input features and the output layer with the activation function f as seen in Figure 2.

As in Figure 2, we employ log function as the nonlinear function *f* because of its promising characteristic. By using a log function as in Equation (2), many natural processes have a history dependent progression in which it begins small and accelerates to some point and then approaches to a saturation point over input features:

(2) $${\mathrm{Cost}}_{i}=\log(X_{i}\cdot W^{T})$$

**Figure 3.**
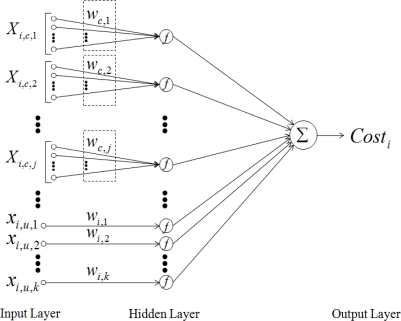
Link cost function represented in PCNN.

Now, let’s discuss the connectivity of the inputs in the network. In Figure 2, all inputs are fully connected to the function. It is just like black-box style connection which is commonly used in NN. However, we intuitively know that some inputs are highly correlated to generate the output of the cost function. For instance, if HMRR(*x*_1_) is high then QL(*x*_2_) and RSS(*x*_3_) are high because they are correlated among them. To take this into the consideration, we connect the inputs in the coupled and uncouple connection style according to whether inputs are correlated or uncorrelated to the hidden layer which is the output layer in Figure 2, as seen in Figure 3. There is no weight on the connections between the hidden layer and the output layer. For instance, the connections of *x*_1_, *x*_2_, and *x*_3_ are coupled and that of *x*_4_ is uncoupled in the PCNN.

From Figure 3, our derived cost function can be formularized as Equation (3):

(3) $${\mathrm{Cost}}_{i}=\sum_{j}\log\left(X_{i,c,j}\cdot W_{c,j}^{T}\right)+\sum_{k}\log\left(x_{i,u,k}\cdot w_{i,k}^{T}\right)$$

where *X_i,c,j_* and *x_i,u,k_* are the *j*th coupled(*c*) input vector set and the *k*th uncoupled(*u*) input collected from the *i*th neighbor node respectively. *W_c,j_* and *w_i,k_* are the corresponding weight vector set and weight respectively. For instance, in our inputs, we have one coupled input vector set, [*x_i,_*_1_, *x_i,_*_2_] and only one uncoupled input *x_i,_*_3_. From Equation (3), the optimal performance of the cost function depends on the proper weight vector.

**Figure 4.**
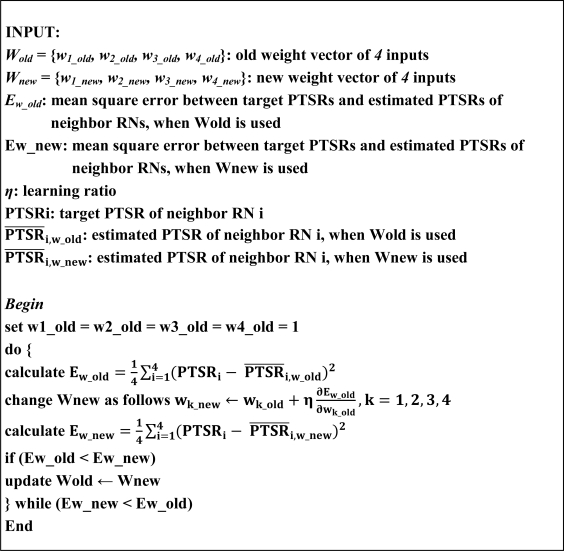
Training algorithm of the cost function.

The optimal performance of the cost function depends on the proper weight vector which can be obtained by training the cost function to reach to the maximum of the packet transmission success ratio (PTSR). To find an optimal weight vector, we imitate the training process [9] for finding the optimal weights in an NN as seen in Figure 4. Each weight in the cost function means the importance of each input for producing the link cost. Thus, each weight can be obtained by weight sensitivity with respect to PTSR.

In general, it is challenging to determine an optimal learning ratio during training. If we choose too large value of *η*, it causes a high convergence speed but it has high possibility of missing the optimal weight values. Too small value of *η* is the reverse of too large a value of *η* where convergence speed is too small but has low possibility of missing the optimal weight values. However, we can determine the learning ratio from exhaustive empirical experiment because there are only four weight metrics in our applications as in Equation (2).

## 4. Performance Evaluation

To validate the performance of our data delivery mechanism in the collection subsystem, we compare the performances of our method with those of Fully Connected NN, (FCNN [14]) and the conventional method. In the conventional method, only one out of the four inputs is used in computing the cost function, *i.e.*, f(*x*_1_), f(*x*_2_), f(*x*_3_), and SSR [15]. SSR (Self-Selective Routing) finds the next node with the smallest number of hops to the destination using the lecture hall algorithm originated in the field of NN. SSR uses the hop distance to select the next forwarding node as input metric and then estimates the hop distance from a node to the destination, using NN technique. From the above rationale, SSR is a kind of the conventional method using hop distance in computing the cost function.

For the construction of the single-hop collection subsystem, 20 nodes are randomly placed in a 500 m × 500 m area. From preliminary experimental results, the optimal learning ratio (*η* = 0.3) is derived to maximize PTSR, as shown in Table 1. Besides, the training time is not issued in the experiments because the NNs are trained within about 5–10 seconds. The training time includes the packet transmission delay and CPU processing time. The link cost function operates in constant time O(1) and repeats n × 1/*η* times. Thus, CPU processing time depends on the number of inputs, n. In this case n is only four, which is very small compared to other NN applications with tens or hundreds of inputs. The CPU processing time for training the link cost function is very small which is negligible in our application.

We tested our mechanism using the NS-2 simulator. We use the log-normal model to model radio propagation environment. A node sends a HELLO message for every 100 milliseconds. IEEE 802.11 standard is used as the MAC layer. The transmission range of a node is 250 m, and the total simulation time is 360 sec. Each node maintains a single queue of packets from all flows passing through the node. We use an exponentially weighted moving average of the instantaneous queue length as a measure of congestion. The average queue length is updated whenever a packet is inserted into the queue.

**Figure 5.**
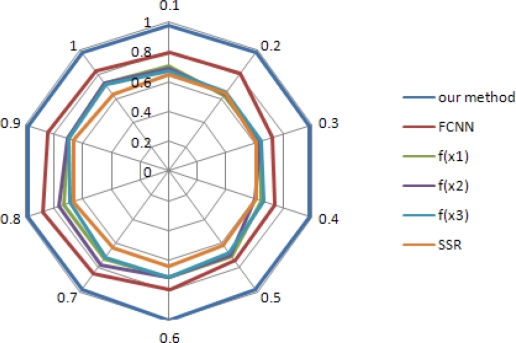
PTSR with varying HMRR using Gaussian distribution with *N*(0, *σ_1_*).

To analyze the effect of collision on PTSR, we vary the probability of packet collision, using Gaussian distribution with zero mean and standard deviation (*σ_1_*) as seen in Figure 5. Methods using the NNs are more robust than the conventional method, irrespective of the degree of packet collision. Method using our method improves the performance compared to the FCNN and the conventional method, by about 22% and 45% respectively.

**Figure 6.**
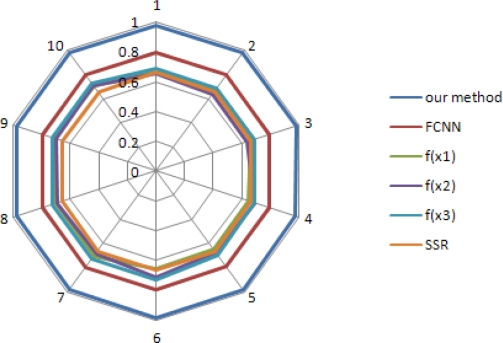
Packet transmission success ratio with varying QL using Gaussian distribution with *N*(0, *σ_2_*).

Figure 6 shows the PTSR obtained by varying the QL, using Gaussian distribution with zero mean and standard deviation (*σ_2_*). It shows the effect of network congestion on PTSR. The results indicate that method using our method delivers more packets than the FCNN and the conventional methods, by about 17% and 51%.

**Figure 7.**
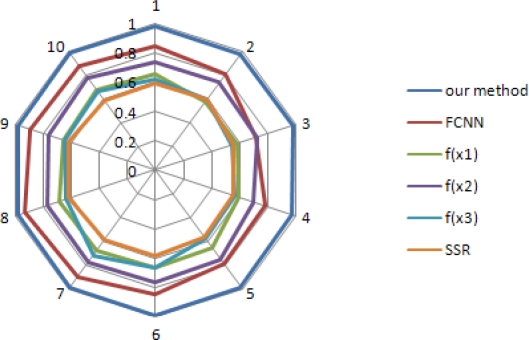
Packet transmission success ratio with varying RSS by adding log normal random fading with *N*(0, *σ_3_*).

Figure 7 is the PTSR obtained by varying the RSS in the shadowing propagation model. For varying, we add the log normal random fading with zero mean and standard deviation (*σ_3_*). From the figure, we can see that method using our method is more robust despite of dynamic random fading and also improve PTSR (about 23% and 43%), compared to the FCNN and the conventional method.

From the above experimental results, we can conclude that the data delivery mechanism using our method improves PTSR without the burden of large overhead occurred during training our method.

## 5. Conclusions

We propose a reliable data delivery mechanism for power quality monitoring system, by modeling an optimal data delivery function. There are several contributions in our method: we have designed the collection subsystem to deliver power quality data from pole transformers to the substations. In order to deliver the power quality data reliably, we developed a reliable link cost function using the neural network concept. For the development, we applied the input type of input feature in connecting them in the neural network, which is an important factor for improving the performance. Also, we showed the feasibility of our method from comparison of our method with the FCNN and the conventional method. From the comparison, we can conclude that the performance of our method is better than those of the conventional methods with respect to PTSR.

There are two approaches for efficient data delivery; one is power control and the other is the link cost function. In this paper, we focused on the development of link cost function to solve the data delivery problem. Since the power control approach can be one of solutions to solve the problem, we will consider the approach as another research direction. Also, we need to extract more input features to meet the requirements and characteristics of applications and systems such as ITS (Intelligent Transportation System) and wireless mesh network.

## Figures and Tables

**Table 1.** Packet transmission success ratio to determine the optimal learning ratio (*η*).

η	**0.1**	**0.2**	**0.3**	**0.4**	**0.5**	**0.6**	**0.7**	**0.8**	**0.9**	**1.0**
PCNN	0.928	0.943	0.979	0.913	0.902	0.893	0.853	0.801	0.797	0.763
FCNN	0.757	0.769	0.792	0.749	0.744	0.734	0.706	0.660	0.611	0.599
